# An Examination of Visitor and Tiger Activity Between Two Zoo Tiger Groups

**DOI:** 10.1002/vms3.70286

**Published:** 2025-02-28

**Authors:** Chaonan Li, Zahrah Alostad, Chad Crittle, Eduardo J. Fernandez

**Affiliations:** ^1^ School of Animal and Veterinary Sciences University of Adelaide Roseworthy South Australia Australia; ^2^ Adelaide Zoo Adelaide South Australia Australia

**Keywords:** behaviour, environment, Sumatran tigers, tigers, visitors, welfare, zoo

## Abstract

Sumatran tigers (*Panthera tigris sumatrae*) are currently listed as critically endangered on the IUCN Red List. In modern zoos, the need to balance the welfare of tigers while maintaining visitor interest presents a significant challenge. The aim of this study was to assess the behaviour of five Sumatran tigers housed in two separate groups and habitat areas (male housed alone; female with three cubs), as well as in relation to visitor crowd size and sound intensity (dB) near the habitats. Five categories of behaviour (active, inactive, social, repetitive and other) were observed and analysed. The most frequent behaviour observed for all tigers was inactivity. The only significant differences in tiger behaviours observed were lower social behaviours in the male tiger housed alone compared to both the female tiger and the cubs. Sound intensity, which was measured in the public viewing area and was positively correlated with crowd size, was significantly louder near the female and cub habitat area compared to the male alone habitat area. A near significant larger crowd size for the female/cub habitat area was observed as well. Crowd size and sound intensity were positively correlated for both tiger groups/habitats, as was crowd size and repetitive behaviours for both the female tiger and the cubs. Results are discussed in the context of animal welfare of captive Sumatran tigers, as well as the promotion of the coexistence of captive tigers and zoo visitors in a manner that supports visitor education/entertainment.

## Graphical abstract

Top left sentence: Two tiger groups were compared for both tiger activity (male alone to female with cubs) and visitor activity (crowd size and sound intensity).

Bottom left sentence: Few differences were observed between the two tiger groups (male alone compared to female with cubs), or between the female and the cubs.

Bottom right sentence: Crowd sizes were near significantly smaller at the male alone habitat (*p* = 0.0647), and sound intensity was significantly less (*p* < 0.001).

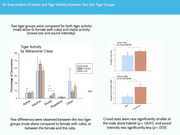

## 1 Introduction

### 1.1 Background and Research Field

Sumatran tigers (*Panthera tigris sumatrae*), the wild subspecies of tiger, are exclusively native to the Indonesian island of Sumatra and are now classified as critically endangered according to IUCN Red List (Goodrich et al. 2022). Sumatran tigers, along with other tiger subspecies, are often kept in zoos, where major efforts have been led to care for and conserve the species (Tilson et al. 1997). Although modern zoos prioritize animal welfare and conservation as two of their primary goals, the education and entertainment of the visitors remain equally important (Fernandez et al. 2009; Learmonth et al. 2021; Sherwen et al. 2018). This includes conservation efforts, including educating visitors about and providing visitors with opportunities to engage in conservation action (Godinez and Fernandez 2019; McNally et al. 2024; Spooner et al. 2023). Therefore, it becomes imperative to understand the impact of visitors on zoo‐housed tigers, the impact of viewing zoo tigers on visitors and the ability to use this information to devise strategies that can bring more visitors without compromising the welfare of zoo tigers.

**Summary**

- Although tigers are considered popular zoo animals, little is known about the impact of visitors on tiger activity and vice versa.
- We examined differences in crowd size, sound intensity (dB) and tiger activity between two different exhibit areas.
- The male tiger housed alone was significantly less social compared to the female tiger and the cubs, with the latter female tiger and cubs housed together.
- Sound intensity (dB) was significantly louder in the public viewing area of the female and cub tiger exhibit compared to the public viewing area of the male tiger exhibit.
- Understanding the relationship between animal and visitor activity can aide in adjusting variables that impact both.

Research about the impact of visitors on the welfare of zoo animals shows increasing evidence that visitors can have various effects on zoo animals (Fernandez and Chiew 2021; Fernandez and Sherwen 2024; Williams et al. 2023). Animal responses to the visitors have been classified by Hosey (2000) into three categories: (1) stressful or negative, (2) no effect or neutral and (3) enriching or positive. These diverse results may be potentially influenced by individual animal factors, such as species activity and previous experience, and environmental variables, such as enclosure design and enrichment (Fernandez et al. 2023; Queiroz and Young 2018; Sherwen and Hemsworth 2019). For instance, Quadros et al. (2014) found considerable individual variation in the impact of noise on the behaviour of a variety of mammals. Likewise, environmental factors can impact zoo felid behaviour. For example, De Rouck et al. (2005) observed a significant increase in pacing behaviour among female captive tigers when they had an unknown tiger as their neighbour. Likewise, Goldsborough (2017) found an increase in tiger stereotypies and aggression during high visitor density times, and Suárez et al. (2017) found that several species of felids spent more time hiding, resting, or away from visitors when the zoo was open to the public.

### 1.2 Study Purpose

The primary objective of this research was to examine the differences in behaviours of five Sumatran tigers residing in two separate groups and habitats (one adult male housed alone; one adult female housed with three cubs), while concurrently evaluating the impact of visitor‐related factors (crowd size and sound intensity [dB]). These factors encompassed a direct measure of visitor activity: crowd size, and an indirect measure of visitor activity: noise intensity (measured in A‐weighted decibels; dBa [herein referred to as dB]). We therefore measured the correlation between the above factors, primarily focused on differences between the two tiger groups (male alone and the adult female tiger with cubs) and the two visitor activity measures.

## 2 Materials and Methods

### 2.1 Subjects and Setting

**FIGURE 1:**
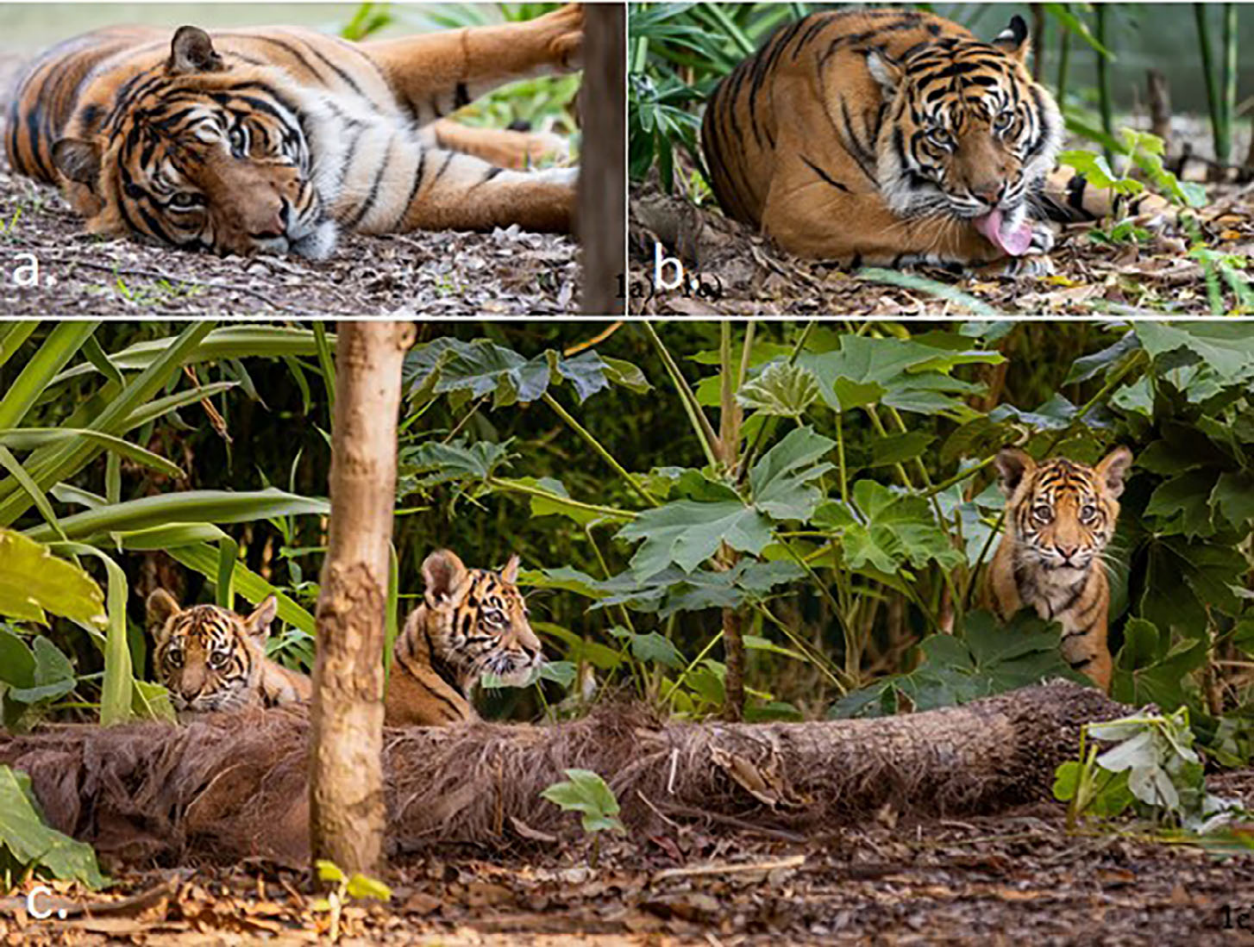
The five Sumatran tigers in Adelaide Zoo: the male tiger, Kembali (a), the female tiger, Delilah (b); and their three cubs (c). *Source*: Photo credit to Adelaide Zoo.

Prior to implementation, the study was approved through the University of Adelaide Animal Ethics Committee (S‐2023‐016). The study was conducted with five Sumatran tigers located at the Adelaide Zoo (Figure 1). A 9‐year‐old adult male tiger (Kembali) was housed alone, and a 5‐year‐old adult female tiger (Delilah) was housed with her three cubs all less than 1 year in age. Notably, these two exhibited areas were contiguous, featuring a transparent iron gate separating the two areas. The adult male habitat area was approximately 18 m × 27 m, whereas the female with cubs habitat area was approximately 32 m × 30 m. Both habitats were directly viewable by the public through a large fenced and windowed areas.

### 2.2 Method and Procedure

Data collection involved making observations of tiger behaviour, recording crowd numbers and collecting sound level measurements. ZooMonitor, a web programme commonly utilized in collecting observations of animal behaviours (Wark et al. 2019), was used to collect the data, allowing simultaneous recording of observations between crowd size, tiger behaviour and sound level (dB)s. Behaviours recorded were determined by a simplified ethogram established prior to the first data collection (see Table 1), which highlighted five key categories of behaviour typically used in zoo ethograms, including active (A), inactive (I), social (S), repetitive (R) and other (O). All categories coded were mutually exclusive, and the final other category included both out of sight and non‐standard responses (those not fitting under one of the previous four categories), thereby making the ethogram exhaustive as well. Although the other category could include non‐standard responses, this was never utilized, with all recorded other category responses meeting the criteria of a tiger being out of sight. In addition, although social behaviours are often limited for tiger species, we maintained its inclusion, as cubs with a mother tiger were present, as well as the ability to compare differences between a solo‐housed male tiger who had limited social contact ability.

**TABLE 1:** Sumatran tiger ethogram, depicting five different behavioural categories (social, repetitive, active, inactive and other), accompanied by the definitions and examples of the behaviour.

Behavioural category	Definition
Social behaviour (S)	Physical contact with another tiger (e.g., paw or mouth contact). Tigers could still make contact through the fenced barrier between the two habitats
Repetitive behaviour (R)	Stereotypical behaviours less commonly seen outside of captivity (e.g., pacing or rocking)
Active behaviour (A)	Any movement‐based behaviour (e.g., locomotion or foraging)
Inactive behaviour (I)	Resting or otherwise non‐movement behaviour (e.g., resting or standing)
Other behaviour (O)	Any time when the tiger(s) were not visible to observers, or any other behaviours not listed in the ethogram (e.g., out of sight or urinate/defecate)

Instantaneous (pinpoint) scan samples (Altmann 1974; Bateson and Martin 2021; Brereton et al. 2022) of 60 s were used to record hour‐long sessions. The numbers of visitors were also recorded every 60 s, with the exact number of visitors recorded up to 21 visitors (excess of 21 visitors were marked as ‘>21’, which provided a finite counting cutoff and rarely occurred). Finally, sound intensity (measured in A‐weighted decibels; dBa) was recorded using Mengshen decibel meters and at a fixed location for each of the two tiger habitats every 60 s (one dB pinpoint sample was taken and recorded). Sessions were collected in front of one of the two habitat areas, so that any given session either focused measures collected in relation to the solitary housed male or socially housed female coded independently of the three cubs coded together (not individually identified). When coding the tiger cubs, all three responses of the three cubs were simultaneously coded and averaged together for the session. Two observers (authors C.L. and Z.A.) completed all observations. Because only two observers and only a limited number of variables were recorded, no interrater reliability was measured. Instead, both observers were trained to collect data via the ethogram and other recorded variables by the supervisor (author E.J.F.), first via video and visual demonstration, then live at the zoo. A total of 44 1‐h sessions were collected between 10:00 and 16:00 in the day (average male habitat: 12:49; average female and cub habitat: 12:31) and between the 18 August 2023 and 10 October 2023.

### 2.3 Statistical Analysis

SigmaStat, version 12.5 (Systat Software Inc. San Jose, CA, USA) was used to create all the graphs and run all the statistical analyses. All data were averaged by the 60 intervals (pinpoints) per session. Only the observations with more than 30 intervals were selected for analysis (sessions were occasionally terminated early do to unforeseen and uncontrollable factors, such as a tiger being moved out of their habitat). This left 22 sessions for the solitary male and 18 sessions for the female/cubs, with four total sessions removed that had less than 30 intervals. Shapiro–Wilk tests were used to test for normality, with only the crowd size data being normally distributed. Therefore, a Kruskal–Wallis test was used to compare differences in the behaviours of the male, female and cubs, a *t*‐test was used to compare differences in crowd size between the two tiger groups, and a Mann–Whitney *U*‐test was used to compare differences in sound intensity (dB) between the two habitat areas. Finally, Spearman's rank correlations were used to test the relationships between crowd size, sound intensity (dB) and the five classes of behaviour for all three tiger groups.

## 3 Results

### 3.1 Activity Budgets

**FIGURE 2:**
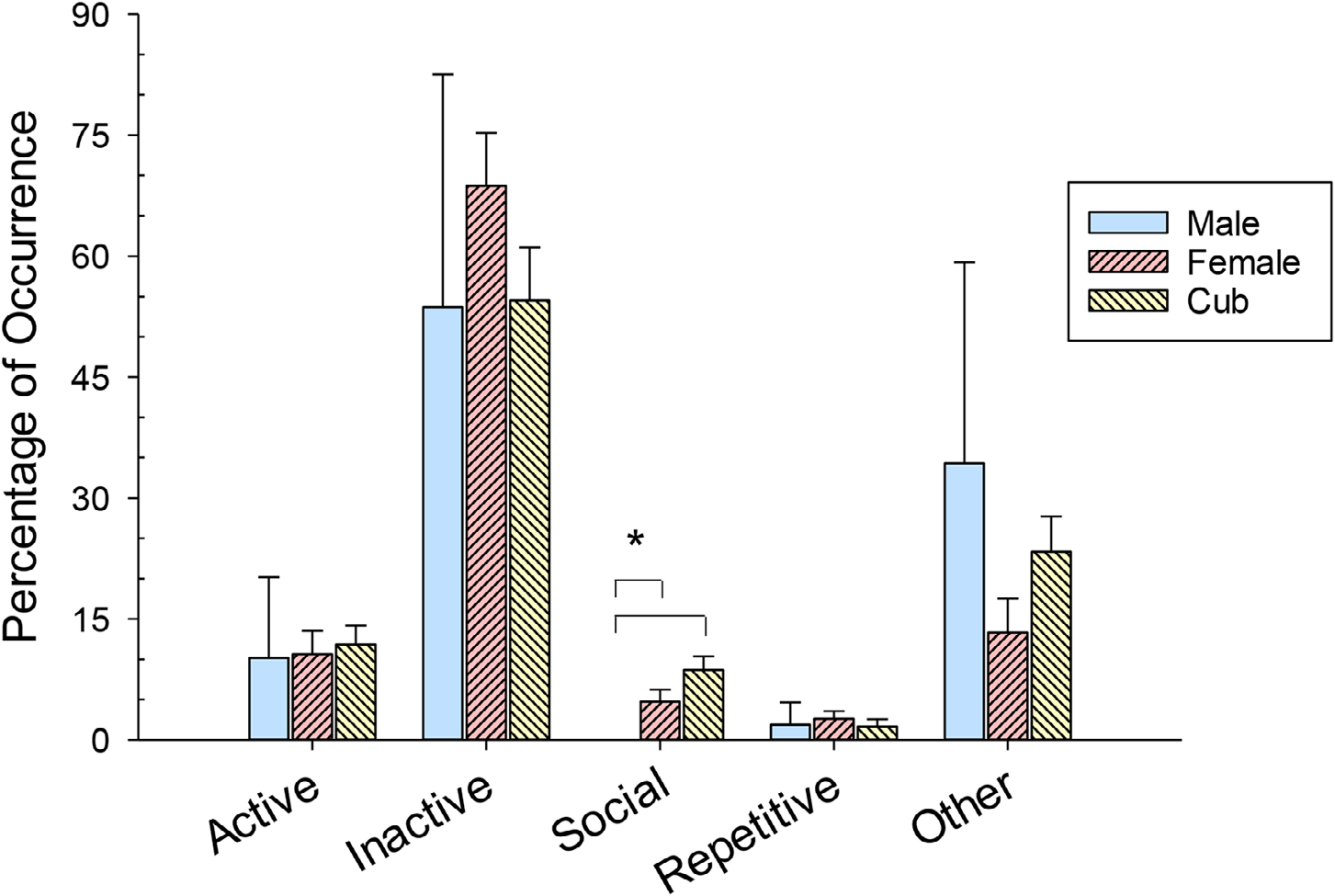
The average percentage of occurrence per session (with standard error of the mean bars) for the five classes of behaviour (*x*‐axis) across the three tiger groups. Solid bars with a ‘*’ represent significant differences (*p* < 0.05). The other category exclusively comprised out of sight behaviours.

Figure 2 shows the difference in the average occurrence for the five classes of behaviour between the male tiger, the female tiger and three cubs.

Inactive was the most frequent class of behaviours for all three tiger groups (male, *M* = 53.7, SE = 28.9; female, *M* = 68.7.0, SE = 6.6; cubs, *M* = 54.5, SE = 6.6). Active behaviours remained relatively constant among the three groups of tigers, ranging from 10% to 12% of all behaviours recorded (male, *M* = 10.2, SE = 10.0; female, *M* = 10.6, SE = 2.9; cubs, *M* = 11.8, SE = 2.3). The only significant difference observed for the ethogram was in differences between social behaviours (*H* = 4.555, df = 2, *p* < 0.001). Post hoc (Dunn's Method) comparisons showed that the female tiger (*p* < 0.003; *M* = 4.7, SE = 1.5) and the cubs (*p* < 0.001, *M* = 8.7, SE = 1.7) were significantly greater than that of the male, who was an individually housed tiger that had limited access to engage in social behaviours. Repetitive behaviours occurred less than 3% for all observed tigers (male, *M* = 1.9, SE = 2.8; female, *M* = 2.6, SE = 0.94; cubs, *M* = 1.7, SE = 0.9). Finally, average other behaviours occurred variably across the tiger groups, with the male engaged in other 34.3% (SE = 25.0), the female engaged in other 13.3% (SE = 4.2) and the cubs engaged in other 23.3% (SE = 4.4).

### 3.2 Crowd Size

**FIGURE 3:**
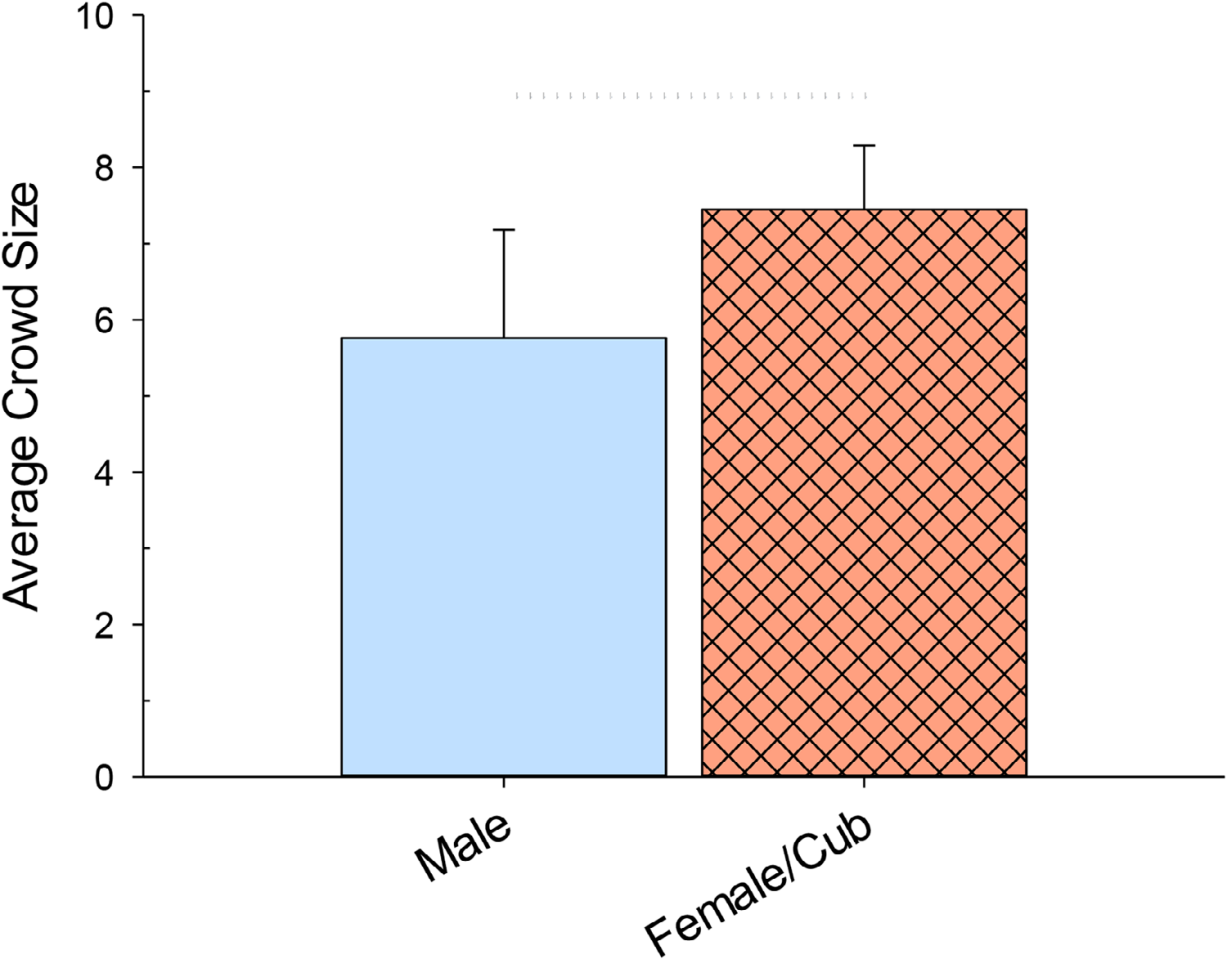
The average visitor crowd size per session (with standard error of the mean bars) for the two different tiger groups/habitats (male alone and female with cubs). The dotted line above represents a difference that was near significance (*p* < 0.10).

Figure 3 shows the difference in crowd size between the two habitat areas (male alone and female with cubs).

The average crowd size at the male alone habitat area was 5.8 visitors (SE = 1.4), compared to 7.5 visitors (SE = 0.8) at the female with cubs habitat area. Nonetheless, this difference between the two habitats was not significant, but rather, near significant (*t* = −1.902, df = 38, *p* = 0.0647).

### 3.3 Sound Intensity

**FIGURE 4:**
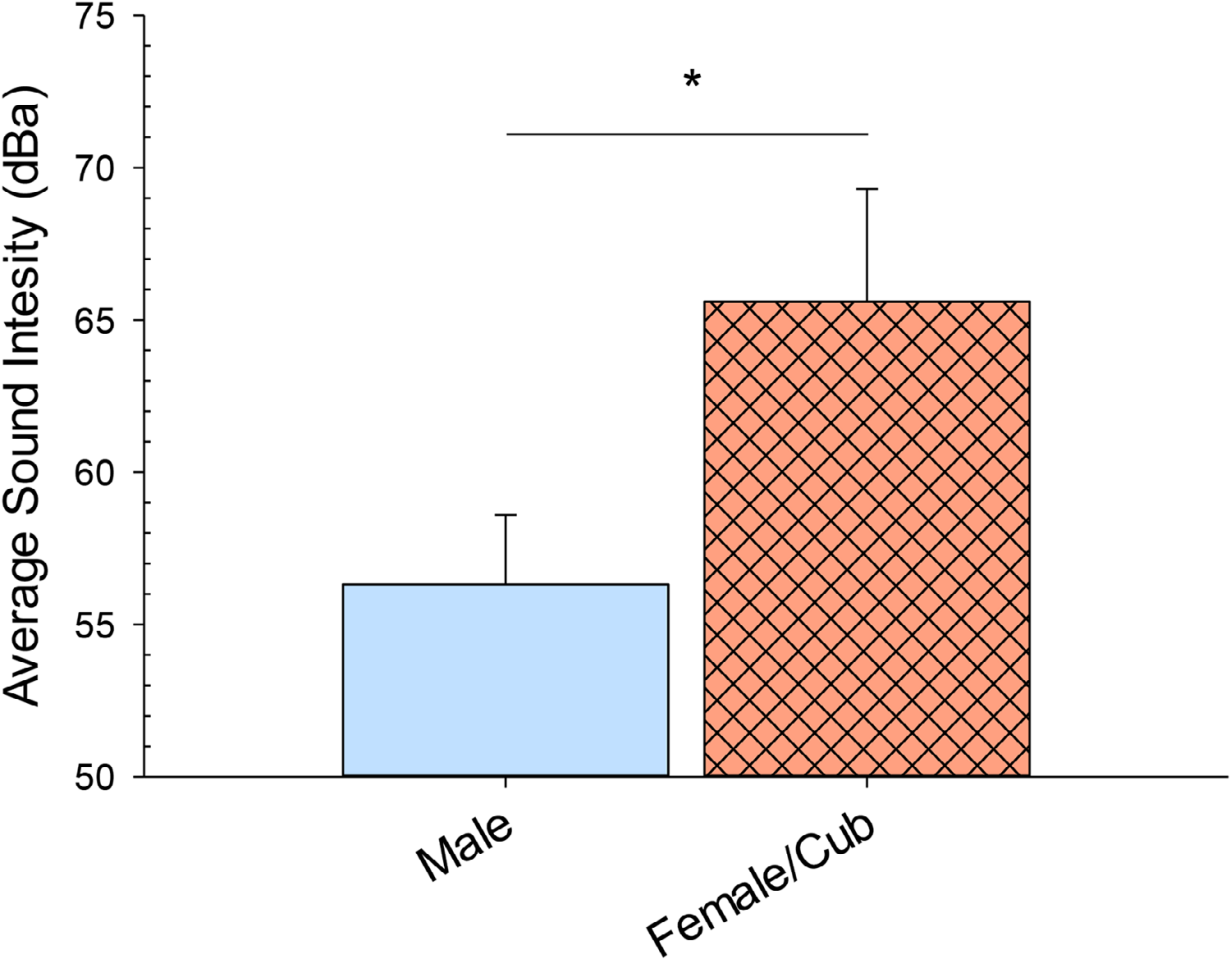
The average sound intensity per session (dBa; with standard error of the mean bars) for the two different tiger groups/habitats (male alone and female with cubs). The solid line with star above represents a significant difference (*p* < 0.05).

Figure 4 shows the difference in sound intensity (dB) between the two habitat areas (male alone and female with cubs).

The average sound intensity at the male alone habitat area was 56.3 dB (SE = 2.3), compared to 65.6 dB (SE = 3.7) at the female with cubs habitat area. This resulted in a significant difference between the two habitat areas (*U* = 73.0, *n*1 = 18, *n*2 = 22, *p* < 0.001).

### 3.4 Crowd Size, Sound Intensity and Tiger Activity Correlations

A total of four significant correlations were found between crowd size, sound intensity and any of the three tiger groups’ behavioural classes. Moderate‐to‐strong positive correlations were observed between crowd size and sound intensity (dB) for both the male alone habitat (*r*_s_ (20) = 0.840, *p* < 0.001) and the female with cubs habitat (*r*_s_ (16) = 0.629, *p* = 0.005). In addition, moderate positive correlations were observed between crowd size and repetitive behaviours for both the female tiger (*r*_s_ (16) = 0.484, *p* = 0.041) and the cubs (*r*_s_ (16) = 0.618, *p* = 0.006).

## 4 Discussion

The main objective of this research project was to examine differences in tiger behaviour in relation to social housing conditions and tiger groups, as well as visitor variables, including crowd size and noise intensity. Upon initial evaluation of the results, it was found that the tigers in both groups/habitats spent most of their time inactive, and that only social behaviours differed significantly between the two groups. However, this was a direct result of our physical interaction requirement for our social behaviour definition, which limited the social interactions possible for the male tiger housed alone (they were only possible for the male tiger through the gate between the two habitats).

The primary differences observed were in relation to visitor variables, specifically the sound intensity (dB) recorded at the two habitat areas. Although not specified as crowd volume, the sound intensity recorded for all sessions was almost certainly a result of visitor activity, with visitors creating more noise (e.g., speaking more and louder) near the habitat area with the adult female tiger and her cubs. In addition, there were moderate‐to‐strong positive correlations found between sound intensity and crowd size. Finally, although not significantly different, it was observed that on average, crowd sizes were larger around the female with cubs habitat area than the male alone habitat area. Similar results have been observed for differences in habitat areas and visitor activity, including differences between the individuals and number of animals exhibited (Margulis et al. 2003; Northey et al. 2022).

It is essential to take into consideration the factors that may have resulted in the disparity between crowd size among the two different groups and habitat areas. Causes such as the presence of cubs might have been responsible for attracting more visitors and therefore observing higher sound intensity levels around the habitat area. In addition, due to the female tiger and cubs’ habitat area being located adjacent to the orangutan habitat area, this may have led to more visitors being present in that area at any given moment. The location of the sound intensity recordings, while reliably consistent for each area, may have also resulted in differences between the two area's recordings. Finally, the correlational nature of these results allow us to draw few conclusions about the causal nature of the observations recorded.

Tigers held in zoos and similar environments are known to display increased levels of stereotypical behaviours such as pacing (Bashaw et al. 2007). In our study, we found moderate positive correlations between crowd size and the occurrence of repetitive behaviours for both the adult female tiger and cubs housed together. Similar findings have been observed for zoo jaguars, where visitors will maintain both large crowd sizes and visitor lengths of stay, whereas the animals engaged in pacing or similar stereotypies (Godinez et al. 2013). In contrast, zoo visitors will often report poor perceptions of such activities, both in the Godinez et al. study and in response to having observed tigers pace (Miller 2012). Therefore, we cannot assume a potential benefit to the visitor experience due to the positive correlation between increased stereotypies and increased visitor crowd sizes or lengths of stay; as such visitor activity can increase, whereas visitors still maintain a poor perception of the animals. In addition, it is possible that the correlation between repetitive behaviours and crowd size that we observed was the result of the tigers pacing as a result of the increased crowd size.

Wild adult tigers tend to have more solitary behaviour in the wild and partake in less amounts of social behaviour, and their time is mostly spent within their own territory (Szokalski et al. 2012). Consideration of typical tiger behaviours observed in both captivity and the wild should be considered when planning for zoo tiger habitats, particularly when considering how to socially house tigers and how visitors might respond to such housing arrangements. How animals are housed in zoos is known to impact both their welfare and the zoo visitor experience (Fernandez and Sherwen 2024; McNally et al. 2024; Williams et al. 2023). Greater consideration for how tigers respond to both other tigers and visitors, as well as what visitors experience when viewing zoo tigers, should facilitate our ability to plan for future zoo tiger habitats.

## Author Contributions

**Chaonan Li and Zahrah Alostad**: conceptualization, data curation, formal analysis, investigation, methodology, writing – original draft, writing – review and editing (equal). **Chad Crittle**: project administration, resources. **Eduardo J. Fernandez**: conceptualization, data curation, formal analysis, investigation, methodology, project administration, software, supervision, writing – original draft, writing – review and editing.

## Ethics Statement

The authors confirm that the ethical policies of the journal, as noted on the journal's author guidelines page, have been adhered to and the appropriate ethical review committee approval has been received through the University of Adelaide (S‐2023‐016).

## Conflicts of Interest

The authors declare no conflicts of interest.

### Peer Review

The peer review history for this article is available at https://www.webofscience.com/api/gateway/wos/peer‐review/10.1002/vms3.70286.

## Data Availability

Data are available from the corresponding author upon request.
